# The changing face of kuru: a personal perspective

**DOI:** 10.1098/rstb.2008.0085

**Published:** 2008-11-27

**Authors:** John D. Mathews

**Affiliations:** School of Population Health, University of MelbourneCarlton, Victoria 3010, Australia

**Keywords:** kuru, Creutzfeldt–Jakob disease, cannibalism

## Abstract

The epidemic of kuru is now known to have been transmitted among the Fore by ritual consumption of infected organs from deceased relatives. As cannibalism was suppressed by government patrol officers during the 1950s, most transmission had ceased by 1957, when the kuru research programme first commenced. As predicted in the 1960s, the epidemic has waned, with progressive ageing of kuru-affected cohorts over the years to 2007. The few cases seen in the twenty-first century, with the longest incubation periods, were almost certainly exposed as children prior to 1960. Although the research programme had almost no role in bringing the kuru epidemic to an end, it did provide important knowledge that was to help the wider world in controlling the later epidemics of iatrogenic and variant Creutzfeldt–Jakob disease and bovine spongiform encephalopathy.

## 1. Introduction

In their invitation letter for the 2007 meeting, John Collinge and Michael Alpers wrote: ‘The epidemic of kuru may not be entirely over but the end is certainly in sight, with at most only one death expected during the year. This is a cause for celebration’. The end of kuru celebrates the end of a tragedy for the Fore, albeit one that is still deeply felt by those who lived through the epidemic. We join all those at the London meeting in thanking Michael Alpers and John Collinge for imagining that such a meeting could ever be possible, and for ensuring that so many Fore were able to attend and to contribute to its success through their personal stories.

The scientific story of kuru has been summarized recently (see Collinge *et al*. (2006) and the papers included in this issue). A detailed account of the early years of kuru research, highlighting Carleton Gajdusek's personal history and involvement, will soon be published (Anderson in press). Table 1 gives an abbreviated timeline of early theories about kuru.

## 2. Student beginnings

I was still at high school in 1957 when Gajdusek and Zigas wrote their first paper on kuru; in the years that followed, Carleton was the main player in an all-consuming programme of research to understand its aetiology. My small part in the story began in 1962 when as a medical student attending a Walter and Eliza Hall Institute (WEHI) seminar I heard Macfarlane Burnet speak about kuru; needless to say, it fired my youthful imagination. Burnet was a leading virologist and immunologist, and won the Nobel Prize in 1960 for the theory of acquired immunological tolerance. Gajdusek worked at WEHI, where Burnet was the Director, before starting the kuru project (see Anderson (in press) for a detailed account of their interactions). Mac Burnet encouraged me to read the published papers, and I corresponded with Michael Alpers in 1963. Michael and I did not meet, however, until 1969, because when I first went to Papua New Guinea in 1964 Michael had moved to the National Institutes of Health to work with Gajdusek.

Burnet was also corresponding with P. R. J. Burch, a radiation physicist in Leeds who had developed mathematical models to explain the age-specific incidence of autoimmune and other chronic diseases (Burch 1963). Burch went on to fit one of his models to the age incidence of kuru, and Burnet showed me Burch's manuscript; unfortunately, Burch had misinterpreted the available data, thereby invalidating the model. After this was pointed out, Burch responded quickly by fitting a different model to correctly interpreted data. I do not think Burch's paper on kuru was ever published, but the experience left me with a healthy respect for the pitfalls of modelling. The irony is that in recent years I have taken up mathematical modelling in my own research (Mathews *et al*. 2007).

Burnet also spoke to me about the work of Shirley Glasse (now Lindenbaum), an Australian anthropologist working with Robert Glasse on the ethnography of kuru (Glasse 1964). They had obtained details of the sorcery that was believed by the Fore to be the cause of kuru. However, the work of the Glasses also suggested that kuru was of relatively recent origin, that it spread from place to place and that it might even be transmitted by cannibalism (Glasse 1962, 1963; Lindenbaum 1979). Although Michael Alpers has recently introduced an alternative term, transumption, since the word ‘cannibalism’ can be seen to be pejorative, I have used the older term, since it was the one in use at the time.

Shirley was in Melbourne in 1964 and, over tea in her parents' home, she showed me the genealogies of kuru-affected families that she and Robert had collected during their fieldwork in 1961–1963. With Shirley's approval, I later obtained copies of the genealogies, and thought about ways to quantify the data that they had collected.

At the end of 1964, after medical finals, it was arranged that I should travel to Okapa and spend six weeks looking at the epidemiology of kuru with Dick Hornabrook, a Queen Square-trained neurologist from New Zealand, who was oversighting the Australian research effort on kuru. Dick and Fay Hornabrook made me feel very welcome; I was enchanted by the landscape and the Fore people, fascinated by the disease, but troubled by the impact it was having upon them (figure 1); there was nothing that western medicine could do to help, and even the attempts to take care of the many kuru orphans at the Awande Lutheran Mission were fraught with problems. Dick told me that by 1964 there were fewer cases of kuru among young children than had been reported by Gajdusek & Zigas (1957); similar trends were noted by Alpers & Gajdusek (1965). I was encouraged to connect the genealogical information from the Glasses with the emerging changes in epidemiology.

Early in my residency year at the Royal Melbourne Hospital, the *Lancet* published my quantitative analysis of the Glasse genealogies and recent changes in kuru incidence (Mathews 1965). The Glasse data showed very clearly that kuru was of very limited time depth, particularly in South Fore villages. Families from Purosa could recall many deaths from causes other than kuru in the years before 1920, whereas the earliest deaths from kuru did not appear until *ca* 1930. Informants also suggested that kuru was first noticed at Uwami, near Awande, and had taken some 20 years to reach Purosa. The obvious conclusion was that with such a recent origin for kuru, the particular genetic theory proposed by Henry Bennett (Bennett *et al*. 1958, 1959) was probably wrong. Furthermore, the march of kuru was consistent with a slowly spreading infectious disease, with the possibility of vertical spread from mother to child to account for the ‘early onset’ cases in children; an implication was that the incubation period could be as long as the age of the children affected (i.e. at least 4 years). Importantly, the age of the early onset cases seemed to be rising from year to year (figure 2). Burnet wrote an appendix to my *Lancet* paper in which he developed the theory that kuru was caused by a hepatitis-like virus, introduced into the Fore in the early decades of the twentieth century (Burnet 1965). That was the closest that I ever came to being a co-author on a paper with Burnet, or for that matter with any Nobel Prize winner!

Hadlow (1959) had pointed out the similarity of kuru and scrapie; this observation, together with the Glasses' research suggesting that kuru was spreading like a slowly transmissible disease, led Gajdusek and colleagues to proceed to inoculation experiments in primates. Their persistence was vindicated in May 1965 when the first chimpanzee (Georgette) developed symptoms of kuru, just 20 months after intracerebral inoculation with brain material from a human patient (Gajdusek *et al*. 1966).

## 3. Full-time in Papua New Guinea

In early 1966, I was employed by the Public Health Department to succeed Dick Hornabrook as coordinator of Australian kuru research in Papua and New Guinea. Coralie and I travelled to Okapa via Goroka, with our son in our arms, and a daughter on the way (our two daughters were born in Goroka Hospital in 1966 and 1967); we quickly settled into a busy life marked by field trips into a beautiful but challenging environment (figure 3), by getting to know the friendly Fore people and by the intriguing social interfaces between local people, researchers, the Australian administrators and the many missionaries in the area. For extended periods when no other medico was available, I was also responsible for medical and surgical care at the Okapa Hospital; at such times, I was grateful for the old-style teaching from our obstetrics professor in Melbourne about how to deal with an obstructed labour or death *in utero*! With the wisdom of hindsight, I sometimes shudder at the other procedures that we were forced to undertake when it was not possible to evacuate patients to Goroka.

We also had many scientific visitors, and Coralie can tell many stories about looking after them amidst our growing family, helping with emergency operations in the Okapa Hospital and providing assistance on field trips and at autopsies.

My appointment in New Guinea had been arranged by Burnet but, as pointed out by Warwick Anderson, who has scrutinized the early correspondence and records, I was naively unaware of the political and personal tensions between Gajdusek, Burnet and the other players. However, from my perspective, there was no alternative but to cooperate with Gajdusek; any other course would have seemed unethical. We welcomed Gajdusek and his colleagues into our home when they visited; we were excited by his fund of stories, his drive and his intellect; and we were inspired to work hard to meet his requirements for kuru data, additional biological samples from kuru patients and unaffected Fore, and film footage. With Ray Spark, who joined in 1967, and a group of Fore assistants, we documented every new case of kuru and its clinical course, and obtained genealogical information and stories about the spread of kuru from family informants. Copies of all our fieldwork notes, clinical examinations and notes, genealogies and kuru stories, and most of the biological samples and film collected were sent to Gajdusek's laboratory in Bethesda.

Owing to continuing requests for biological samples (blood and cerebrospinal fluid), and autopsy material, we felt that we were at risk of putting undue pressure on patients and their families to obtain the necessary consent. We spent many hours explaining our research, and its objectives, and in building rapport, but it was never possible to be certain that consent, when it was given, was free of implied duress in a cross-cultural environment that was still very colonial. A chapter of my MD thesis (Mathews 1971) was devoted to the ethics and propriety of our research with the Fore.

## 4. Epidemiological studies supporting the cannibalism hypothesis

Gajdusek and Zigas provided us with access to epidemiological details of all known kuru cases in *The village file*, for the era from 1957 to 1963; we added in the cases seen by Hornabrook and myself, and checked the details against census data to complete the records through to early 1968; subsequent work by many people, but most particularly by Michael Alpers, has tracked the epidemic through to its dying stages, 50 years after the first cases were reported in the scientific literature (Alpers 2008). During our time among the Fore, I used the available epidemiological data, together with my own genealogical data and ‘kuru stories’, to further test the specific predictions made by the genetic theory, by the ‘slow virus’ theory with horizontal and vertical transmission and by the cannibalism theory. A paper in the *Papua New Guinea Medical Journal* (Mathews 1967*a*) provided additional support for the epidemic spread of kuru. It also showed that in the South Fore, there had been almost no cases of kuru in children born since 1954; the average age of kuru cases was rising year by year, suggesting that vertical transmission, or transmission to young children, had ceased in the 1950s (figures 2 and 4). This was hard to accommodate with the original genetic theory, but was consistent with cannibalism or other forms of transmission to women and children. An obvious implication was that as the kuru epidemic matured, the average age of cases would continue to increase, with an eventual decline in overall incidence. Nevertheless, in that paper (Mathews 1967*a*), the genetic theory could be rescued as a possible explanation for some of the differences in the incubation period, coincidentally anticipating the effect of the codon 129 polymorphism (Collinge *et al*. 2006).

The implications of horizontal transmission (to explain kuru in adults) and vertical transmission (to explain kuru in children) were further developed in another *Lancet* paper (Mathews 1967*b*). For sibships born before 1940, there was a birth order effect for kuru in males and in females developing kuru before the age of 20 years; the higher risk in later-born siblings was consistent with an acquired agent, but did not discriminate between direct transmission to the child and transmission through the mother. Brothers concordant for kuru tended to die at a similar age, suggesting exposure at a similar age rather than at a similar time. Kuru occurred at an earlier average age in women in later-born cohorts, suggesting that in the early stages of the epidemic females were exposed at a common time, rather than at a common age; in the mature epidemic, the reverse may have applied. The ages of male kuru cases, putting an upper limit on the lengths of presumptive incubation periods, ranged from 4 or 5 to 40 or more years.

This 1967 paper argued that it was possible to accommodate all the epidemiology with the idea that there was horizontal transmission to women, and vertical transmission to offspring, together with an increase in the incubation period over the maturing epidemic owing to stochastic variation, selection and/or differential genetic susceptibility. Nevertheless, that model had complex features. Robert Glasse's original hypothesis (Glasse 1963, 1967), that kuru was transmitted through the practice of eating relatives who had died from the disease, seemed simpler. A major attraction of that theory was that as men rarely participated at cannibal feasts, there was a ready explanation for horizontal transmission being largely confined to women. Young children were exposed because they accompanied their mothers and also ate kuru-contaminated tissues, including brain; this provided a simple explanation for ‘vertical transmission’ or early onset kuru. In a paper with Robert Glasse and Shirley Lindenbaum (Mathews *et al*. 1968), we showed that the cannibalism theory was supported by a wealth of epidemiological and ethnographic evidence; there were also consistent stories about named individuals who had taken part in ritual feasts and subsequently died themselves from kuru (table 2).

I later wrote up unpublished details of kuru fieldwork in my MD thesis (Mathews 1971) and in a monograph edited by Dick Hornabrook (Mathews 1976). Those data allowed kuru incubation periods to be estimated from the stories of known exposures of named individuals who subsequently developed kuru (table 2). Common exposure period analysis for pairs of affected relatives provided similar estimates, and supported the argument for post-natal transmission to males in early childhood. By assuming that affected brothers may have had a common exposure, it was possible to deduce a mean minimum age of exposure of 1–6 years for males, a mean minimum incubation period of 3–6 years and a mean maximum incubation period of 10–14 years for the era (1930–1967) covered by the data collected (Mathews 1971). Clearly, the incubation period has increased subsequently with the ageing of kuru-affected cohorts.

Perhaps the most important epidemiological observation was the disappearance of childhood kuru, with an increase, year by year, in the ages of people affected by kuru (figure 2). The simplest interpretation was that new transmissions of kuru were stopping in the 1950s, when government patrols suppressed cannibalism. The ageing of the kuru-affected cohorts, together with the lengthening of the incubation period in the post-transmission epidemic, can explain the observed increases in age of kuru onset. Indeed, as Michael Alpers reports in his paper (Alpers 2008), none of the victims of kuru in the years since 1968 were born after 1960, when transmission had almost certainly ceased. This, together with the waning of the epidemic, provides the best proof that the epidemic was indeed transmitted by cannibalism, albeit with incubation periods that could be as short as 4 years, but occasionally in excess of 40 years.

## 5. CJD, oral transmission and Carleton's Nobel Prize

Carleton's laboratory achieved the successful transmission of Creutzfeldt–Jakob disease (CJD) to chimpanzees in 1967. He was out bush at the time the telegram came to Okapa, announcing the breakthrough, so it was handed to me. Coralie and I recognized it as the harbinger of the Nobel Prize, which was to follow in 1976. For reasons that are unclear, Gajdusek was often sceptical about kuru being transmitted by consumption of contaminated tissues; when pressed, he would argue that the agent was more likely to have been transmitted by accidental inoculation through wounds or lesions of the skin or gastrointestinal tract. Nevertheless, by 1980, his laboratory had shown that kuru and CJD could both be transmitted orally to spider monkeys, apparently in the absence of lesions that could provide a parenteral portal of entry (Gibbs *et al*. 1980).

During the 1980s, the prion story began to emerge, showing that what had originally been thought of as a slow virus was actually an aberrant form of a normal protein (Prusiner 1982; Collinge *et al*. 2006). The prion diseases were classified as transmissible spongiform encephalopathies (TSEs). The later epidemics of bovine spongiform encephalopathy (BSE) and variant CJD (vCJD) were to show, in a very dramatic way, that prion-induced diseases other than kuru were also orally transmissible.

## 6. Looking back

In a coda to this personal tale, as Deputy Chief Medical Officer for the Australian Government from 1999 to 2004, I was belatedly drawn back into the TSE story owing to a need to provide policy advice to protect Australia from both BSE and vCJD. One of our strategies was to call on the expertise of Michael Alpers and Colin Masters, two Australians who had made major contributions to the understanding of the transmissible encephalopathies. The TSE committee on which we served together allowed me to revisit the kuru story, learn about prions and consolidate friendships from the past.

Of course, I had ended my active involvement in kuru research at a very much earlier stage when we moved in 1968 from Okapa back to Melbourne, to allow me to take a position in the Clinical Research Unit at WEHI. Over the intervening years, I have realized what a great privilege it was to be involved in the kuru story, to interact with Burnet and Gajdusek, Michael Alpers and Shirley Lindenbaum, and the many others who visited us in New Guinea. I corresponded with Robert Glasse, but sadly we never met; he died in 1993. I corresponded with other researchers while in Okapa, some of whom I later met at Gajdusek's Nobel Prize celebration in Bethesda, or by happenstance in later life. Above all, it was a privilege to work with the Fore people, to have made friends with many of them and to have played a small part in explaining what had caused their tragic disease.

An irony is that although the Fore have seen at first hand the very same evidence that has led science to attribute kuru to cannibalism, most Fore people, even today, still believe that it was caused by sorcery. It is also humbling to remember that the intervention that eventually prevented kuru (i.e. the suppression of cannibalism by government patrol officers) was almost complete by the time that research on the problem commenced. Although medical science contributed very little to the end of kuru, the understanding triggered by research on the kuru problem has been of great value in helping to control the subsequent epidemics of BSE and vCJD in the wider world. We should pause to thank the Fore for that, as well as the many talented researchers who have provided us with such deep understanding of the TSEs in the years since 1957!

## Figures and Tables

**Figure 1:**
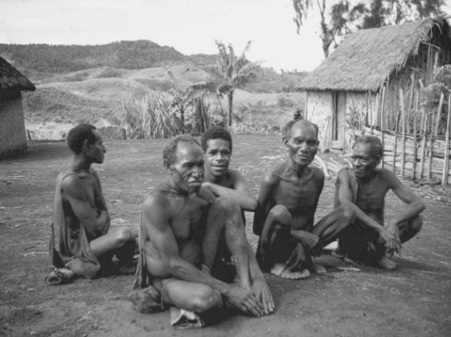
In the 1950s and 1960s, the kuru epidemic killed approximately 25% of the female population in the South Fore; in some villages, there were few female survivors of marriageable age, leaving many orphans and many men without wives.

**Figure 2:**
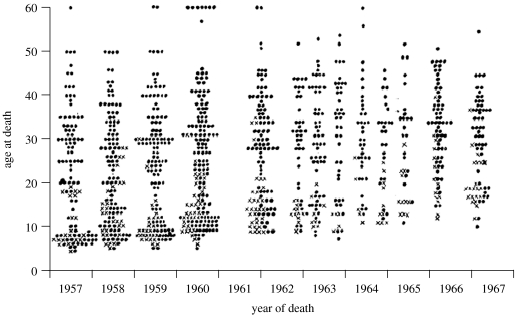
Kuru deaths by age in the South Fore from 1957 to 1967 (crosses, male deaths; dots, female deaths). It can be seen that ‘early onset’ kuru was disappearing; there was a progressive increase in the minimum age of kuru patients with each year that passed.

**Figure 3:**
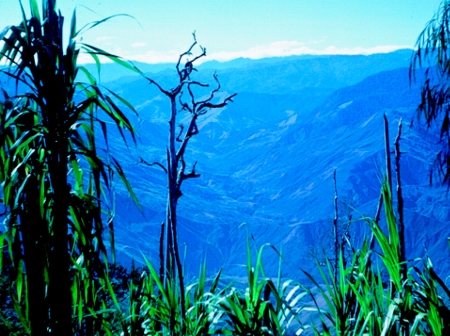
Beautiful but challenging terrain, looking down the Lamari Valley from the South Fore.

**Figure 4:**
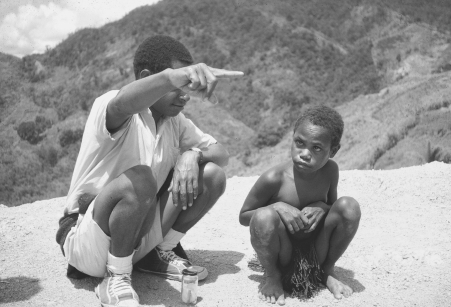
The youngest case of kuru seen in 1967: a girl aged 10 years from a remote village.

**Table 1:** Timelines in early research on kuru.

year	significant event
1951–1953	Ronald and Catherine Berndt record first accounts of kuru sorcery
1951–1955	first reports of kuru from government patrol officers
1955–1956	first cases of kuru examined at Goroka and Kainantu Hospitals
1956	Dr Vin Zigas sends blood samples and a brain to Melbourne
1957 March	Dr Carleton Gajdusek starts fieldwork with Zigas
1957–1958	Bennett, Rhodes and Robson suggest a genetic aetiology for kuru^a^
1959	Hadlow notes that kuru pathology is similar to scrapie
1961–1963	Robert and Shirley Glasse report on recent time depth and spread of kuru, and suggest that cannibalism might be involved in transmission
1965 May	first transmission of kuru to chimpanzee by inoculation
1967	first transmission of CJD to chimpanzee by inoculation
1980	first oral transmission of kuru to spider monkeys

a It was suggested that an autosomal gene was dominant in females, causing late onset female kuru in heterozygous women, and recessive in males, causing early onset kuru in homozygotes of both sexes.

**Table 2:** Early kuru deaths at villages A and B: presumptive incubation periods. (*Notes*. (i) Data from Mathews (1971). (ii) Village and personal names recorded in the field notes but not published in order to protect confidentiality. (iii) Probable transmission pathways deduced from informant accounts of specific persons taking part in the mortuary rituals after an earlier kuru death. (iv) Likely incubation periods are subject to obvious ascertainment biases; furthermore, as transmission had ceased by 1960, incubation periods of kuru cases observed at later times range up to 45 years. (v) Similar data were reported subsequently by Klitzman *et al*. (1984). (vi) ?, unknown.)

approximate year of death	person ID		probable transmission from	likely incubation period
*village A*				
1922	1A	young wife (∼14)	?	?
1931	4A	wife (∼22)	1A	9 years
1931	5A	youth (∼11)	1A	9 years
1932	6A	wife (∼34)	1A	10 years
1934	7A	wife (∼19)	1A	12 years
*village B*				
1940	1B	adult wife	?	?
1945	3B	adult wife	1B	5 years
1946	5B	adult wife	?	?
1950	9B	adult wife	5B	4 years
1952	10B	adult wife	1B	12 years
1954	18B	adult wife	1B	14 years
1955	19B	adult wife	5B	9 years
1956	21B	adult wife	1B	16 years
